# Draft Genome Sequences from a Novel Clade of *Bacillus cereus Sensu Lato* Strains, Isolated from the International Space Station

**DOI:** 10.1128/genomeA.00680-17

**Published:** 2017-08-10

**Authors:** Kasthuri Venkateswaran, Aleksandra Checinska Sielaff, Shashikala Ratnayake, Robert K. Pope, Thomas E. Blank, Victor G. Stepanov, George E. Fox, Sandra P. van Tongeren, Clinton Torres, Jonathan Allen, Crystal Jaing, Duane Pierson, Jay Perry, Sergey Koren, Adam Phillippy, Joy Klubnik, Todd J. Treangen, M. J. Rosovitz, Nicholas H. Bergman

**Affiliations:** a Biotechnology and Planetary Protection Group, Jet Propulsion Laboratory, California Institute of Technology, Pasadena, California, USA; b National Biodefense Analysis and Countermeasures Center, Ft. Detrick, Maryland, USA; c Department of Biology and Biochemistry, University of Houston, Houston, Texas, USA; d Department of Medical Microbiology, University of Groningen, University Medical Center Groningen, Groningen, The Netherlands; e Lawrence Livermore National Laboratory, Berkeley, California, USA; f Johnson Space Center, Houston, Texas, USA; g Marshall Space Flight Center, Huntsville, Alabama, USA

## Abstract

The draft genome sequences of six *Bacillus* strains, isolated from the International Space Station and belonging to the *Bacillus anthracis*-*B. cereus*-*B. thuringiensis* group, are presented here. These strains were isolated from the Japanese Experiment Module (one strain), U.S. Harmony Node 2 (three strains), and Russian Segment Zvezda Module (two strains).

## GENOME ANNOUNCEMENT

Among the six *Bacillus cereus sensu lato* strains reported here, three U.S. Harmony Node 2 isolates (ISSFR-3F, ISSFR-9F, and ISSFT-23F) and one Japanese Experiment Module isolate (JEM-2) were sequenced and assembled with both Illumina MiSeq and PacBio RSII sequence data. The remaining assemblies, including two Russian isolates, were sequenced and assembled with only MiSeq data. The MiSeq runs yielded on average 24 to 54 million 300-bp reads (from 1,402× to 3,093× average coverage), while PacBio yielded 4,000 to 116,000 reads (from 7× to 202× average coverage) (Table 1). Due to the extremely high coverage (>1,000×), Illumina MiSeq reads were randomly down-sampled to 100× using an estimated genome size of 5.3 Mb, resulting in an average of 1.2 to 1.5 million paired-end reads per isolate. Next, the down-sampled reads were assembled using iMetAMOS (1) with IDBA_UD and SPAdes (2). IDBA_UD was selected as the best assembly for all six isolates. Low confidence bases within the selected IDBA_UD (3) assemblies were masked out by mapping all reads to the assembled contigs and detecting conflicting variants with FreeBayes (4). The PacBio reads were assembled following the methods described by Berlin et al. (5) with Celera Assembler version 8.3rc1 and polished with Quiver (6). A second round of polishing was performed post-Quiver using the available MiSeq data as input to Pilon (7).

**TABLE 1:** *De novo* assembly statistics of the *Bacillus* spp. isolated from the ISS

Isolate no.	ISS module	GenBank accession no.	Sequencing platform	No. of reads	Avg. read coverage (×)	*N*_50_ (Mb)	No. of contigs
ISSFR-3F	U.S.^a^	CP018931	PacBio	116,418	202	5.2	2
ISSFR-9F	U.S.	CP018933	PacBio	15,939	42	5.2	2
ISSFR-23F	U.S.	MSMO00000000	PacBio	8,145	17	0.6	16
JEM-2	Japan^b^	CP018935	PacBio	40,305	80	5.2	2
S1-R4H1-FB	Russian Federation^c^	NBNT00000000	Illumina	4,275,902	242	0.1	329
S2-R3J1-FB-BA1	Russian Federation	NBNR00000000	Illumina	11,266,760	638	0.4	388

a U.S., U.S. Segment Harmony Node 2.

b Japan, Kibo Japanese Experiment Module (JEM).

c Russian Federation, Russian Segment Zvezda Module.

The six International Space Station (ISS) isolates were aligned (NUCmer [8], Parsnp [9]) against members of the *B. cereus sensu lato* group genomes (10). The PacBio assemblies were used for all isolates with sufficient read coverage, and Illumina assemblies were used for the remaining isolates. Based on genome size estimates (5.2 to 5.3 Mb), NUCmer pairwise alignments (>99.9% average pairwise nucleotide identity) and maximum-likelihood phylogenetic placement, all six isolates were found to exhibit a very high degree of similarity.

Finally, due to their high genomic similarity to the *B. anthracis* type strain (>98% average nucleotide identity), all six genomes were examined for evidence of pathogenicity. However, none of the commonly known *B. anthracis* signature elements were identified. Specifically, all six ISS isolates (i) contain the *plcR* (11) ancestral “C” allele, which has been used in large-scale phylogenetic analyses to distinguish *B. anthracis* strains from the rest of the *B. cereus* group; (ii) lack significant hits to pXO1 and pXO2 plasmids; and (iii) are phylogenetically placed outside of the *B. anthracis* clade. Results were consistent with a comparative genomic analysis performed using the Lawrence Livermore National Laboratory Microbial Threat Characterization Pipeline. Altogether, the collective genomic evidence supports the conclusion that the six ISS isolates represent a novel *Bacillus* sp. located within the *B. cereus sensu lato* group.

### Accession number(s).

The complete genome sequences were deposited in NCBI under the accession numbers listed in Table 1 and can be accessed from the NASA GeneLab system (GLDS-64; https://genelab-data.ndc.nasa.gov/genelab/accession/GLDS-64).
